# Spectroscopic Interpretation of PAH-Spectra in Minerals and Its Possible Application to Soil Monitoring

**DOI:** 10.3390/s100403868

**Published:** 2010-04-15

**Authors:** Eun-Joung Ko, Kyoung-Woong Kim, Kihong Park, Ju-Yong Kim, Jiwon Kim, Se-Yeong Hamm, Jung-Hwan Lee, Uwe Wachsmuth

**Affiliations:** 1 Department of Environmental Science and Engineering, Gwangju Institute of Science and Technology, 1 Oryong-dong, Buk-gu, Gwangju 500-712, Korea; E-Mails: ejko@kird.re.kr (E.-J.K.); kpark@gist.ac.kr (K.P.); juyongk@gist.ac.kr (J.-Y.K.); jiwonkim1219@gmail.com (J.K.); 2 Division of Earth Environmental System, College of Natural Science, Pusan National University, Busan 600-735, Korea; E-Mails: hsy@pusan.ac.kr (S.-Y.H.); oathway@pusan.ac.kr (J.-H.L.); 3 Laser Laboratorium Goettingen, Hans-Adolf-Krebs-Weg 1, 37077 Goettingen, Germany; E-Mail: uwachsmuth@llg-gmbh.de

**Keywords:** PAH, fluorescence, spectral structure, contaminant-soil interaction

## Abstract

In order to properly assess the feasibility of using Laser-Induced Fluorescence (LIF) spectroscopy for soil monitoring, the variation of fluorescence intensity due to the heterogeneity and complexity of soil media was investigated. Different soil minerals showed fluorescence spectral structures distinguishable from the contaminants, implying dissimilar interactions or the binding of contaminants on mineral surfaces. More interestingly, solvent and water addition showed different responses in the fluorescence spectral structure showing their effect on the interactions between contaminants and minerals. These results support the claim that the spectral structure contains information on contaminant-mineral interactions; therefore contaminants can be used as a fluorescence probe for these interactions.

## 1. Introduction

In recent years, several research groups have developed fiber optic Laser-Induced Fluorescence (LIF) sensors for online and *in-situ* monitoring of soils and aromatic hydrocarbons in water, soil and air. These systems have several interesting applications, such as field screening of oil contamination in water and soil after environmental accidents, and localization of subsurface oil plumes at contaminated waste sites [1–10]. LIF results are expressed as a function of wavelength, time and intensity. For a given wavelength range, the integrated intensity is easily considered an indicator of the concentration of contaminant. Using this concept, initial *in situ* monitoring techniques, such as SCAPS-LIF and ROST-LIF, can be used to detect the presence of the contaminant as a screening method [4,5].

However, in contrast to LIF applications of monitoring pollutants in water, the heterogeneity and the opacity of soil matrices are still considerable experimental challenges for the qualitative and quantitative interpretation of LIF data [11]. The *in situ* fluorescence response of the LIF to hydrocarbon compounds is sensitive to variations in the soil matrix. For the *in situ* LIF analysis of contaminants in soil, the measured fluorescence intensity can be described by the following general equation [12]:

(1) $$\left[I_{F}(\lambda_{\mathrm{em}})=\mathrm{const}\;I_{0}(\lambda_{\mathrm{ex}})\varepsilon *(\lambda_{\mathrm{ex}})\;\eta_{F}(\lambda_{\mathrm{ex}})f(M)c\right]$$

where the term *const* includes constant and experimental parameters such as detection geometry and detector sensitivity. I_0_(λ_ex_) is the excitation intensity, ε*(λ_ex_) and η_F_(λ_ex_) are effective extinction coefficients and fluorescence efficiencies, respectively, and c is the concentration of the analyte. The influence of various matrix parameters on the LIF signal is expressed by ƒ(M). For a given analyte, when the excitation intensity is kept constant, the above equation can be simplified to express the direct proportionality between LIF signal intensity and analyte concentration:

(2) $$\left[I_{F}(\lambda_{\mathrm{em}})=\underline{m(M)}\;c\right]$$

Therefore, the matrix effect as well as the concentration dependency on the fluorescence should be investigated. Matrix properties that affect LIF sensitivity include soil grain size, mineralogy, moisture content, and surface area. Each of these factors influences the relative amount of analyte that is adsorbed on or absorbed into soil. Only the relative fraction of analyte that is optically accessible at the window of the probe can contribute to the fluorescence signal [4].

The widely varying photophysical properties of analytes, which are present in soil sites of interest and matrices, strongly influence the obtained fluorescence signals. These influential factors change not only the fluorescence intensity, but also the fluorescence spectral signature [13]. This means that if we neglect the soil matrix effect or the interaction (or binding) between contaminant and solid media, merely integrated intensity as a sum parameter is deemed useless as a concentration indicator of the contaminant.

Baretz *et al.* [14] and Reyes *et al.* [15] have studied the change in the fluorescence spectra according to surface loading of PAH on various soil media. The fluorescence spectra revealed that the increasing surface loading of PAH induced excimer emission. Thomas [14] mentioned that Spectral fluorescence of an excited state reports back on the environment of the excited state, *i.e*., on the polarity of the surfaces and the nature of adsorption of the molecule to the surface. These studies provided the first layer surface which can be exposed to laser light was only concerned with the fluorescence.

Therefore, in order to develop a means of characterizing the contaminant and to monitor contaminated sites, this study focused on the nature of the surrounding environment which affects the relative fraction of analyte accessible at the window of the probe.

We investigated how the change of the spectral signature reflected the nature of the surrounding environment by observing factors such as the binding and the interactions between the soil media and contaminants. Factors such as solvent, water and surfactant additions to the soil media were used in describing the changes in the ambient environmental conditions. Those factors can also be used to estimate the state or phase of PAH in soil media comparing the fluorescence spectra of PAH adsorbed on the surface and that dissolved in the surfactant.

## 2. Materials and Method

### 2.1. Contaminant/Soil Mineral

Anthracene and pyrene were selected as representative Polycyclic Aromatic Hydrocarbons (PAHs). Anthracene was selected because of its distinct spectral structure. The PAHs used in this study were purchased from Sigma Chemical Co. Quartz (SiO_2_) and aluminum oxide (Al_2_O_3_) were selected as main soil constituents to represent the different soil surface area types and mineralogies, *i.e.*, soil media type. The quartz was a coarse soil type with a yellowish color and a low surface area compared to the aluminum oxide, which represented the fine soil type, with a white color and a high surface area of about 900 m^2^. To prepare the PAH contaminated soil media, fifty grams of soil mineral was mixed with 10 mL of spiking solution where the PAH was dissolved in dichloromethane. To achieve a homogeneous condition, spiking procedures were thoroughly carried out in several steps. The contaminated soil mineral was placed in the fume hood for three days to evaporate the dichloromethane. At 150 mg PAH/kg dry soil, fluorescence was observed.

### 2.2. Solvent/Surfactant

Cyclohexane and dimethyl sulfoxide (DMSO) were selected to investigate the interaction arising from the differences in polarity between the contaminants and their surrounding environments. These solvents have low and high dielectric constants, and were chosen to represent the polar and nonpolar environments, respectively. The effect of water was also checked because of its presence in soil pores. Soil media with 0, 2.5, 5, 10, 15, and 20% solvent content were also prepared to examine the effects of the solvent content on the LIF spectral signature. HPLC-grade reagents were obtained from Fisher Scientific Co.

A biosurfactant was used to monitor its effect on removing the organic contaminants adsorbed on soil media using the LIF. Rhamnolipid, the biosurfactant used in this study, is comprised of extra-cellular natural substances produced by *Pseudomonas aeruginosa* and has low CMC and nontoxic effects to soil microorganisms. Biosurfactant was introduced to enhance the solubility of hydrophobic organic compounds like PAHs. Rhamnolipid solution was purchased from Jeneil Biosurfactant.

### 2.3. Laser Induced Fluorescence (LIF) System

**Figure 1.**
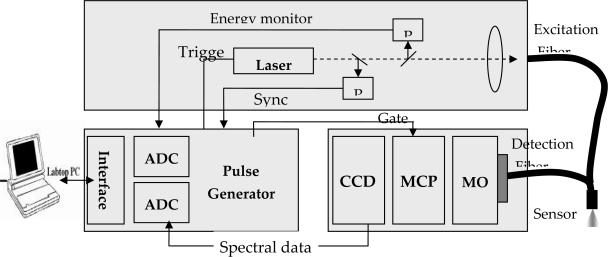
Laser Induced Fluorescence System [PD: Photodiode, MO: Monochromater, MCP: Multichannel plate image intensifier, CCD: CCD camera ADC: Analogue to digital converts].

LIF system used in this study is dealt with. The principal components are the pulsed UV laser as the excitation source, a fiber-optic probe, a detection unit for time and spectrally resolved detection of the fluorescence light, and the control and data acquisition unit. For the excitation of fluorescence, a diode laser pumped neodymium-doped yttrium aluminum garnet (Nd:YAG) laser with a 7 ns pulse duration and the fourth (266 nm) harmonics of the laser was used for excitation of the fluorescence. The detection unit is basically an optical mulit-channel analyzer with a time resolution of 5 ns, which consist of a spectrograph, a gateable image intensifier and a CCD camera (Figure 1).

The light of a pulsed UV-laser is guided through an optical fiber into the observed soil and excites existing pollutants to fluorescence. The fluorescence light is guided back though additional fibers to the detection unit.

### 2.4. Fluorescence Measurements

The fiber-optic probe was dipped into the soil sample, rinsed with ethanol and distilled water, and cleaned with a fine laboratory wiping tissue after each measurement. Typically, the fiber-optic probe carrying UV-laser illuminated a surface area of 20 mm^2^. The fluorescence signal was collected from approximately that spot size. The original spectra from LIF system are time resolved spectra in the total wavelength between 0 and 50 ns and in the wavelength range between 258 and 607 nm. For the entire soil sample, at least five measurements were performed and averaged to avoid any inconsistency due to the heterogeneity of the soil matrix.

### 2.5. Data Analysis

The LIF provides the full Wavelength-Time-Intensity (WTI), which is the time resolved spectra between 0 and 50 ns over the wavelength range 258 to 607 nm. The wavelength coverage of the fluorescent light emitted from the PAHs, and the specific shape of fluorescence spectra were dependent on the PAH itself.

In this study, the total fluorescence intensity and spectral signature were generally obtained by integrating the spectra over the total time interval. Here, the additional time information was only used for the interpretation of surfactant effect on PAH contaminated soil where surfactant and PAH showed their overlapping fluorescence spectra. Time resolved detection of the fluorescent light yields fluorescence decay times. This information makes it possible to simultaneously discriminate between PAHs and surfactant due to their characteristic decay time. The fluorescence intensity, and its relation to the specific wavelength coverage at each respective PAH concentration, were recorded. The total fluorescence intensity was obtained by integrating these spectra for the wavelength coverage of each PAH. Additionally, fluorescence spectral signatures were monitored to assess the different types of PAH binding on the surface of the soil media.

## 3. Results and Discussion

### 3.1. Soil Media (Mineral) Effect

**Figure 2.**
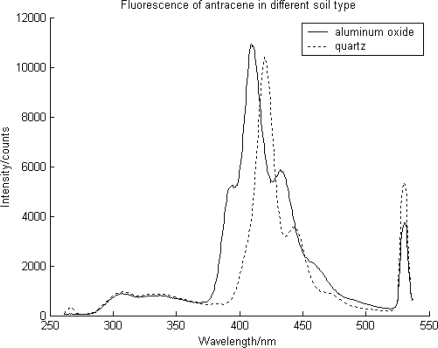
The fluorescence spectra of anthracene contaminated quartz and aluminium oxide.

**Figure 3.**
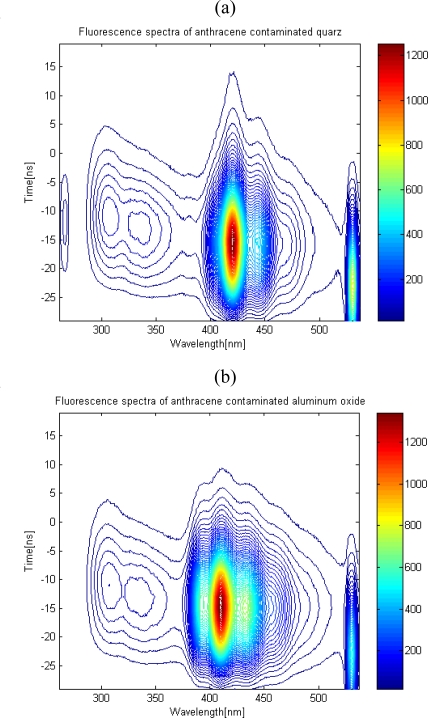
The contour map of the fluorescence of anthracene on different soil mineral type.

The different soil media types were tested to investigate the matrix properties on the fluorescence spectra. At a given concentration of 150 mg PAH/kg dry soil, fluorescence was observed. According to the results, the anthracene on different soil minerals showed different spectral structures, implying different types of binding or interactions between the contaminants and soil mineral (Figures 2 and 3). Likewise, the total fluorescence intensity extracted from the fluorescence curve for the wavelength coverage was affected as well.

For anthracene on quartz, the first peak (bending mode) of fluorescence was deteriorated (Figure 3a); this can be explained as the suppression of bending of molecules due to the strong binding of PAH on quartz compared to the aluminum oxide. On the other hand, aluminium oxide had spectra of somewhat released species on the surface, showing more of an activated bending mode (Figure 3b). These findings implied that different interactions between contaminant moleculea and the environment exist.

**Figure 4.**
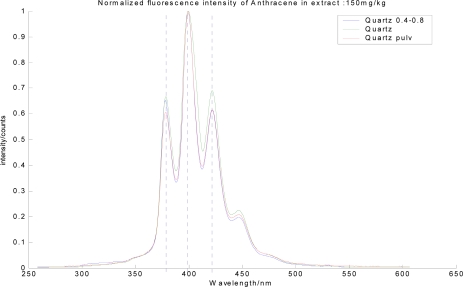
The fluorescence spectra of extract from anthracene contaminated quartz with various size [Quartz 0.4–0.8 mm, Quartz (CRM), Quartz powder].

In order to support this, the fluorescence of extracted anthracene from the contaminated quartz was also observed (Figure 4). Here, quartz of various sizes was used, including quartz Certified Reference Material (CRM), quartz 0.4–0.8 mm and quartz powder. The results showed that the PAH was not destroyed during the measurement when it is compared to PAH on aluminum oxide and in solvent showing distinct spectra structure.

It also cannot be explained by self absorption due to the high concentration or inner filter effect because of its spectral signature shown in 250–370 nm. This observation implies that the fluorescence spectra signature according to the soil mineral type should be carefully considered when it is used as the contaminant indicator with the expression of relative quantities of PAH present in the soil sites of interest. Additionally, this fluorescence spectra signature of anthracene can be a representative probe, which can detect the variation of the state of contaminants in soil. For example, it is possible to deduce the adsorbed or dissolved state of contaminants in the soil environment by comparing the fluorescence spectra of the contaminant in the soil and the solvent.

### 3.2. Solvent Effect

The polarity of solvent and the local environment have profound effects on the emission spectra of polar fluorophores such as PAHs. Generally, solvent effect was evaluated to determine the polarity of the probe-binding site on the macromolecule. This was accomplished by comparing the emission spectra and /or quantum yields of the fluorophore between when it is bound to the macromolecule and when it is dissolved in the solvents of different polarity. This serves as an explanation in that the different soil media types and surrounding local environment of contaminant show different spectral signatures. It means that the estimation of PAH in the soil media could be performed by comparing the fluorescence spectra of PAH on quartz and in solvent. In order to investigate the possible interaction between PAH and soil media, the fluorescence spectral signatures with the addition of solvent on soil media were observed.

**Figure 5.**
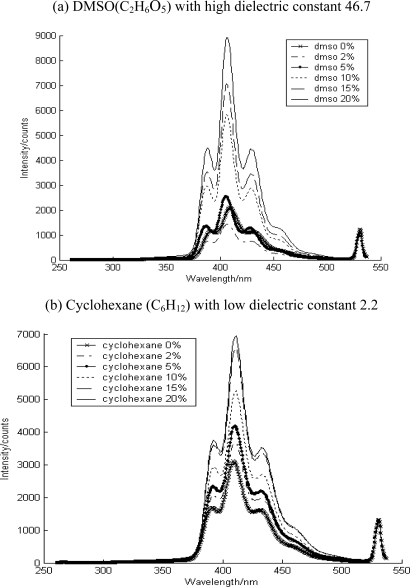
The solvent effect on the fluorescence of anthracene-contaminated aluminum oxide.

**Figure 6.**
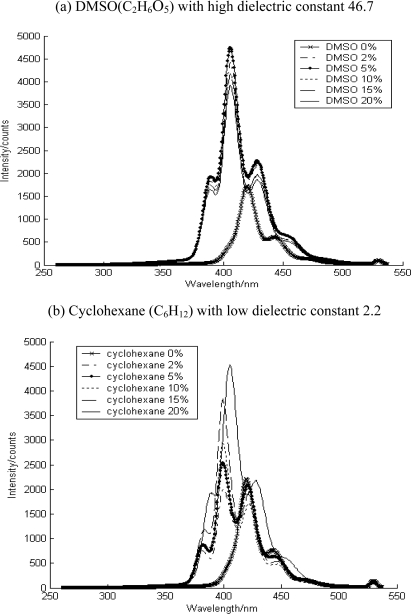
The solvent effect on the fluorescence of anthracene contaminated quartz.

Solvent and water addition showed different responses in the fluorescence spectra, indicating their effects on the interaction between the contaminant and the soil media. The added solvent increased fluorescence intensity in which the contaminant began to dissolve in the additional solvent (Figures 5 and 6). Here, DMSO (C_2_H_6_O_5_) and cyclohexane (C_6_H_12_) were used to describe the different environment around PAH. These solvents with different dielectric constant could affect the solubility of PAHs on soil media, resulting in the change in their fluorescence intensity and spectral signatures.

This figure showed increased PAH fluorescence intensity after addition of both solvents. This implies that as the surface loading of PAH on soil media is increased, the PAH fraction accessible at the window of sensor increased. The fluorescence of aluminum oxide was a sharper structure similar to that in pure solvent. Based on the convoluted structure of aluminium oxide, we can deduce the state or phase of contaminants in soil media; adsorbed species were on the surface or soaked species were in the inner pores. There was no change in the fluorescence spectral signature following solvent addition, as shown in Figure 5. Based on these observations, the contaminants were expected to exist as dissolved species in the solvent or as free molecules and weakly binding species on the surface if adsorbed. In the case of the soil media type such as aluminium oxide, it was deduced to have caused the soaking species to move into the outer space rather than changing the binding on the surface.

As shown in Figure 6, the anthracene on quartz under dry conditions showed typical spectra of adsorbed species on surfaces, implying strong binding on the surface, thus suppressing the bending mode of the molecules of the contaminants. However, as the solvent was added on the soil media, it showed the developed spectral signature. It is possible that as the solvent was added, adsorbed contaminants on quartz surface started to dissolve into the solvent. In other words, the phase of the contaminants in the soil media changed from an adsorbed phase on the surface to a dissolved phase in the solvent. This resulted in the free bending mode of contaminant molecules.

Water had little effect on the state of the contaminants, due to their low solubility in that liquid. Considering the natural water content in soil media, the water effect on fluorescence intensity as well as the spectral signature of the PAHs was assessed.

**Figure 7.**
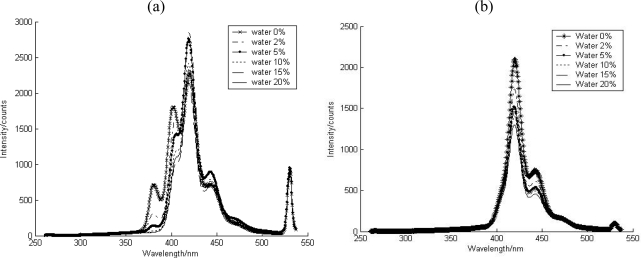
The water effect on the fluorescence of anthracene contaminated (a) aluminum oxide (b) quartz.

Quartz is considered as the first layer surface with low available surface area, and with the continuous addition of water, PAH was displaced from the surface, resulting in a decreased intensity. However, the effects of water result in no change to the spectral signature with increasing water content. The state of the contaminant can be deduced as the adsorbed species on surface in spite of water addition. However, for PAH on aluminium oxide, high water content induced a geometric change of the aluminium oxide, rather than a change in PAH solubility. This yielded a different spectral structure (Figure 7), meaning that the available surface area for contaminant, surrounding environment of contaminant, was changed and that interactions between the contaminant and the soil media was affected.

From these results, we can deduce the role of fluorescence spectra, implying the state of contaminant and its physicochemical interactions with the soil media.

### 3.3. Surfactant Effect

**Figure 8.**
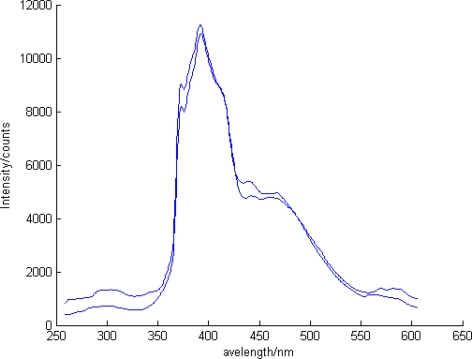
The fluorescence of pyrene in solvent.

**Figure 9.**
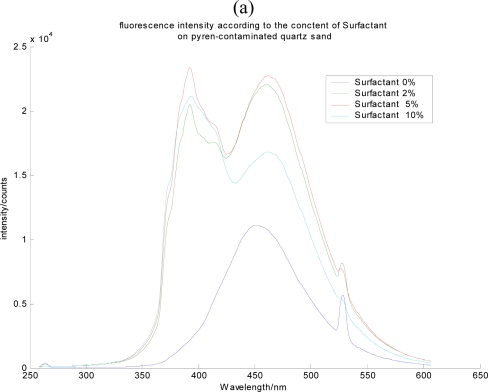

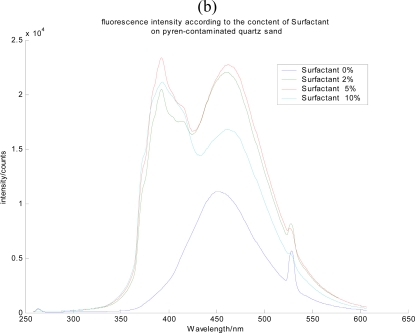
The surfactant effect on the fluorescence of pyrene contaminated (a) aluminum oxide and (b) quartz.

The surfactant effect was investigated to monitor the presence of the contaminants, and to predict the possible state of the contaminant in the soil media during the remediation process monitoring. For organic compounds such as PAHs, the remediation process using surfactant is also commonly performed. Therefore, an accurate appreciation of the surfactant effect on the fluorescence of PAHs was required. Based on the appreciation of the phases or states of contaminants in soil media, the LIF test was performed. Time resolution was applied to distinguish the spectra of the PAH and the surfactant. The fluorescence of PAH in solvent in which PAH existed as a free molecule or dissolved states (Figure 8) and that of PAH from contaminated soil media was compared with the fluorescence of PAH with surfactant addition (Figure 9).

In terms of the peak wavelength and spectral signature of pyrene, the LIF results were interpreted. As the surfactant was added on the soil media, the developing spectral signature of pyrene on the soil media and the subsequent increase in its fluorescence intensity were observed. This supports the fact that as the surfactant is added, adsorbed contaminants on soil media surface starts to dissolve in the surfactant, resulting in the free bending mode of the contaminant molecules. These observations make it possible to monitor the remediation process using surfactants for removing organic contaminant.

## 4. Conclusions

The goal of this study was to develop a means of characterizing the contaminants and any chemical changes taking place by using LIF to monitor the remediation process. For the solvent and surfactant, ways in which the fluorescence spectral signature could be affected were investigated. Different soil minerals showed different spectral signatures, implying different types of binding on the surface of the soil media. Additionally, the change in the environment was also studied using solvent and water. The mixture of these two substances showed different responses in the fluorescence spectra, indicating their effect on the interaction between the contaminant and soil media. The added solvent increased the fluorescence intensity because the contaminant started to dissolve into the added solvent. Though water had a low effect on the fluorescence spectra, due to the contaminant’s low solubility, high water content induced a geometric change in the aluminium oxide. This resulted in different spectra structures, which in turn resulted in the change of the interaction between the contaminant and the soil media. The state of the contaminant can be estimated by comparing the spectral structures of the contaminant in solvent (or water) and solid media with that of the pure solvent on solid media. Therefore, the change of fluorescence spectra of PAH contaminated soils can be correlated with the change of the phase of PAH in soils. This serves as a valuable tool for measuring the progress of the remediation. Additionally, it can support the claim that there is indeed a difference in the removal of the contaminant and the progress of the remediation process.

## Figures and Tables

**Table 1.** The properties of the solvent.

**Solvent**	**Dielectric constant**	**Raman signal**	**Absorption cut off**
Dimethyl sulfoxide C_2_H_6_O_5_(DMSO)	46.68	SO: 273	265
Cyclohexane C_6_H_12_	2.2023	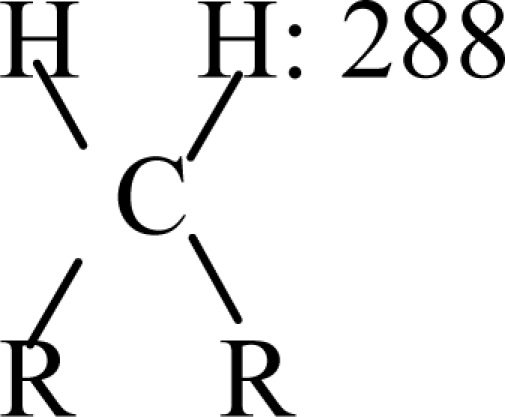	210
Water	78,54	OH: 293–294	-
